# Factors related to outcome of bloodstream infections due to *Candida parapsilosis* complex

**DOI:** 10.1186/s12879-016-1704-y

**Published:** 2016-08-09

**Authors:** Francesco Barchiesi, Elena Orsetti, Patrizia Osimani, Carlo Catassi, Fabio Santelli, Esther Manso

**Affiliations:** 1 Clinica Malattie Infettive; Università Politecnica delle Marche, Azienda Ospedaliero-Universitaria, Ospedali Riuniti Umberto I°- G.M. Lancisi – G. Salesi, Ancona, Italy; 2 Clinica Pediatrica; Università Politecnica delle Marche, Azienda Ospedaliero-Universitaria, Ospedali Riuniti Umberto I°- G.M. Lancisi – G. Salesi, Ancona, Italy; 3 Pediatria, Azienda Ospedaliero-Universitaria, Ospedali Riuniti Umberto I°- G.M. Lancisi – G. Salesi, Ancona, Italy; 4 Anestesia e Rianimazione Pediatrica, Azienda Ospedaliero-Universitaria, Ospedali Riuniti Umberto I°- G.M. Lancisi – G. Salesi, Ancona, Italy; 5 Laboratorio di Microbiologia, Azienda Ospedaliero-Universitaria, Ospedali Riuniti Umberto I°- G.M. Lancisi – G. Salesi, Ancona, Italy

**Keywords:** *Candida paraspilosis* complex, Candidemia, Risk factors, Mortality, Antifungal agents

## Abstract

**Background:**

Although *Candida albicans* is the most common cause of fungal blood stream infections (BSIs), infections due to *Candida* species other than *C. albicans* are rising. *Candida parapsilosis* complex has emerged as an important fungal pathogen and became one of the main causes of fungemia in specific geographical areas. We analyzed the factors related to outcome of candidemia due to *C. parapsilosis* in a single tertiary referral hospital over a five-year period.

**Methods:**

A retrospective observational study of all cases of candidemia was carried out at a 980-bedded University Hospital in Italy. Data regarding demographic characteristics and clinical risk factors were collected from the patient’s medical records. Antifungal susceptibility testing was performed and MIC results were interpreted according to CLSI species-specific clinical breakpoints.

**Results:**

Of 270 patients diagnosed with *Candida* BSIs during the study period, 63 (23 %) were infected with isolates of *C. parapsilosis* complex which represented the second most frequently isolated yeast after *C. albicans*. The overall incidence rate was 0.4 episodes/1000 hospital admissions. All the strains were in vitro susceptible to all antifungal agents. The overall crude mortality at 30 days was 27 % (17/63), which was significantly lower than that reported for *C. albicans* BSIs (42 % [61/146], *p* = 0.042). Being hospitalized in ICU resulted independently associated with a significant higher risk of mortality (HR 4.625 [CI95% 1.015–21.080], *p* = 0.048). Conversely, early CVC removal was confirmed to be significantly associated with a lower risk of mortality (HR 0.299 [CI95% 0.102–0.874], *p* = 0.027). Finally, the type of primary antifungal therapy did not influence the outcome of infection.

**Conclusions:**

Candidemia due to *C. parapsilosis* complex, the second most commonly causative agent of yeast BSIs in our center, is characterized by a non-negligible mortality at 30 days. An early CVC removal is associated with a significant reduced mortality.

## Background

*Candida* is an important cause of bloodstream infections (BSIs) and it is the main agent of invasive fungal infection in hospitalized patients [1, 2]. Although *Candida albicans* is the most common cause of invasive candidiasis, infections due to *Candida* species other than *C. albicans* are rising [3, 4]. In particular, *Candida parapsilosis* complex, which includes *C. parapsilosis* sensu strictu (the most frequent), *Candida orthopsilosis* and *Candida metapsilosis*, has emerged as an important fungal pathogen and became one of the main causes of fungemia in tertiary-care hospitals [5–9]. Although the mortality rate due to this species is generally lower than that reported for other *Candida* species, *C. parapsilosis* complex possesses several distinct features, such as its ability to develop biofilms on intravascular devices, high affinity for parenteral nutrition, and an intrinsic low susceptibility to echinocandins [10–12].

The aim of this study was to analyze the factors related to outcome of candidemia due to *C. parapsilsosis* complex in a single institution over a five-year period.

## Methods

### Study population and data collection

A retrospective observational study of all cases of candidemia was carried out from January 1, 2010 to December 31, 2014 (5-year period) in a single 980-bedded referral University Hospital in Ancona, Italy. A case of *Candida* BSI was defined as a peripheral isolation of *Candida* species from blood culture in a patient with temporally related clinical signs and symptoms of infection. All *Candida* BSIs were identified through the microbiological laboratory database. Data regarding demographic characteristics and clinical risk factors were collected from the patient’s medical records. Appropriate antifungal therapy was considered when an appropriate drug (based on subsequent in vitro susceptibility testing results) with adequate dosage was started within 72 h from the first blood culture performed. Adequate dosage of an antifungal agent was defined according to IDSA 2009 guidelines [13]. Early central venous catheter (CVC) removal was defined a removal of the line within 48 h from drawing blood culture. Mortality was calculated after 7 and 30 days from the occurrence of the episode of *Candida* BSI.

*Candida* species were isolated from blood samples using BacT/ALERT (bioMérieux) and identified with standard techniques. Each of the three species of *C. parapsilosis* sensu lato was identified using the MALDI-TOF Biotyper™ [14]. Antifungal susceptibility testing was performed using the SensitreYeastOne colorimetric plate (SYO) (Trek Diagnostic System) and MIC results were interpreted according to latest species-specific CBPs as established by the CLSI [15].

The present research has been performed in accordance with the ethical standards of the 1964 Declaration of Helsinki and its later amendements. The Institutional Review Board of the Azienda Ospedaliero-Universitaria Ospeadali Riuniti Umberto I°-Lancisi-Salesi granted retrospective access to the data without need for individual informed consent.

### Statistical analysis

Quantitative data were shown as the median with interquartile ranges (Q1–Q3). Qualitative variables were expressed as absolute and relative frequencies. Categorical variables were compared using the χ2 test, whereas Mann–Whitney U test or Fisher exact test were applied for continuous variables. Variables with a *p* ≤0.05 at the descriptive analysis were analyzed by Cox regression. All statistical analyses were performed using the statistical package SPSS for Windows v. 20 (SPSS Inc., Chicago, IL, USA). A *P* value of ≤0.05 was considered to represent statistical significance and all statistical tests were two-tailed.

## Results

Of 270 patients diagnosed with *Candida* BSIs during the study period, 63 (23 %) were infected with isolates of *C. parapsilosis* complex (95 *C. parapsilosis* sensu lato, 3 *C. orthopsilsosis* and 2 % *C. metapsilosis*). This species represented the second most frequently isolated yeast after *C. albicans* (146 [54 %]). The remaining infections were due to *C. tropicalis* (23 [9]), *C. glabrata* (10 [4]) and other *Candida species* (10 [4 %]). Although C. *albicans* represented the most commonly isolated species, its percentage significantly decreased from 68 % to 48 % in the time period considered (*p* = 0.040). On the opposite, there was a significant increase of the percentage of *C. parapsilosis* isolation from 8 % (2010) to 30 % (2014) (*p* = 0.036). The overall incidence rate of BSIs due to *C. parapsilosis* was 0.4 episodes/1000 hospital admissions.

Demographics and clinical characteristics of 63 patients infected with isolates of *C. parapsilosis* complex were compared with those of 146 patients infected with isolates of *C. albicans* and the results are shown in Table 1. Median patients age was 67 years. Male accounted for 56 % of the population. Twenty-five isolates were recovered from patients hospitalized in medical wards, 21 from patients hospitalized in ICUs and 17 from patients hospitalized in surgical wards. The majority of patients (90 %) suffered from multiple comorbidities (range 2 to 4) being the most common cardiovascular (43) and gastrointestinal (33 %) diseases. A total of 51 % of patients underwent a surgical intervention within 30 days from the onset of candidemia. In comparison with *C. albicans*, BSIs due to *C. parapsilosis* were significantly more often associated with the use of CVC (*p* = 0.015). The overall crude mortality at 30 days was higher in patients infected with *C. albicans* than *C. parapsilosis* (42 % *vs* 27 %, respectively, *p* = 0.042).

**Table 1:** Comparison of epidemiological, clinical characteristics and outcome of patients with BSIs due to *Candida parapsilosis* and *Candida albicans*

Variables	Patients with BSIs due to:		
	*C. parapsilosis* (*n* = 63)	*C. albicans* (*n* = 146)	*P* value^*a*^
Age (years), median (IQR)^*b*^	67 (43–76)	51 (66–77)	0.133
Neonates (<3 months)	1 (2)	5 (3)	0.465
Elderly (>65 years)	32 (51)	72 (49)	0.844
Male sex, *n (%)*	35 (56)	91 (63)	0.358
Ward			
Internal Medicine, *n (%)*	25 (40)	52 (36)	0.448
Surgery, *n (%)*	17 (27)	32 (22)	
Intensive Care Unit, *n (%)*	21 (33)	62 (43)	
Comorbidities, *n (%)*	57 (90)	137 (94)	0.387
Chronic pulmonary diseases, *n (%)* ^*c*^	9 (14)	11 (8)	0.127
Haematological malignancy, *n (%)*	1 (2)	8 (5)	0.203
Cardiovascular diseases, *n (%)* ^*d*^	27 (43)	67 (46)	0.685
Neurological diseases, *n (%)* ^*e*^	10 (16)	24 (16)	0.919
Gastrointestinal diseases, *n (%)* ^*f*^	21 (33)	43 (29)	0.576
Diabetes mellitus, *n (%)*	8 (13)	28 (19)	0.254
Solid tumors, *n (%)*	15 (24)	43 (30)	0.403
Chronic renal failure, n (%)	7 (11)	12 (8)	0.504
Previous surgery (<30 days)*, n (%)*	32 (51)	67 (46)	0.514
Gastrointestinal surgery, *n (%)*	8 (13)	19 (13)	0.950
Cardiovascular surgery, *n (%)*	13 (21)	28 (19)	0.807
Other surgery, *n (%)* ^*g*^	12 (19)	24 (16)	0.646
Central venous catheter, *n (%)*	56 (89)	127 (87)	0.702
BSI CVC-related, *n (%)*	36 (64)	57 (45)	0.015
Other devices, *n (%)* ^*h*^	57 (90)	129 (88)	0.653
Previous invasive procedures (<72 h), *n (%)* ^*i*^	16 (25)	44 (30)	0.486
Parenteral nutrition, *n (%)*	43 (68)	105 (72)	0.592
Immunosuppressive therapy, *n (%)* ^*j*^	18 (29)	56 (34)	0.174
Neutropenia, *n (%)*	4 (6)	7 (5)	0.644
Septic shock, *n (%)*	2 (3)	12 (8)	0.180
Prior antibiotic therapy, *n (%)*	58 (92)	139 (95)	0.370
Previous antifungal therapy (<30 days), *n (%)*	5 (8)	21 (14)	0.195
Concomitant bacteriemia, *n (%)*	15 (24)	40 (27)	0.588
Other coinfections, *n (%)* ^*k*^	39 (62)	89 (61)	0.897
Appropriate antifungal therapy*, n (%)* ^*l*^	38 (60)	92 (63)	0.712
Primary azole therapy	41 (65)	77 (53)	0.098
Primary echinocandin therapy	7 (11)	34 (23)	0.041
Primary polyene therapy	1 (2)	3 (2)	0.820
None	14 (22)	32 (22)	0.961
Overall mortality, *n (%)*	17 (27)	61 (42)	0.042
Early mortality (days 1–7)*, n (%)*	4 (6)	24 (17)	0.049
Late mortality (days 8–30), *n (%)*	13 (21)	37 (25)	0.464

^a^Comparisons between groups were performed using Wilcoxon rank sum test for quantitative variables and Chi-Square test (or Fisher Exact Test when expected frequencies were less than five) for qualitative variables

^b^IQR, Interquartile range

^c^Chronic pulmonary diseases include asthma, chronic bronchitis, emphysema and lung fibrosis

^d^Cardiovascular diseases include heart failure, ischemic heart disease, endocarditis and arrhythmia

^e^Neurological diseases include Parkinson’s disease, Alzheimer’s disease and paralysis

^f^Gastrointestinal diseases include Crohn’s disease, ulcerative colitis, chronic pancreatitis and gallbladder stones

^g^ Other surgery includes plastic surgery, thoracic surgery, orthopaedic surgery, urological surgery and neurosurgery

^h^ Other devices include urinary catheter, surgical drainage, cutaneous gastrostomy and tracheostomy tube

^i^Previous invasive procedures include endoscopy and positioning of any device

^j^Immunosuppressive therapy include corticosteroids, calcineurin inhibitors and monoclonal antibodies

^k^Other coinfections include bacterial and/or fungal infections in sites other than blood

^l^Appropriate antifungal therapy was considered when the appropriate drug with adequate dosage was started ≤ 72 h from the first blood culture performed

Factors related to outcome of 63 patients with BSIs due to *C. parapsilosis* are reported in Table 2. Older age (*p* = 0.046), being hospitalized in ICU or in medical wards rather than in surgical wards (*p* = 0.033), being recently diagnosed with a cardiovascular disease (*p* = 0.006), and the lack of an early CVC removal (*p* = 0.003) were all factors associated with a significant higher probability of death at the descriptive analysis. On multivariate analysis, being hospitalized in ICU resulted independently associated with a significant higher risk of mortality (HR 4.625 [CI95% 1.015–21.080], *p* = 0.048). Conversely, early CVC removal was confirmed to be significantly associated with a lower risk of mortality (HR 0.299 [CI95% 0.102–0.874], *p* = 0.027).

**Table 2:** Outcome of 63 patients with BSIs due to *Candida parapsilosis* complex considered in this study

Characteristics	30-day outcome		
	Survival (*n* = 46)	Death (*n* = 17)	*P* value^*a*^
Age (years), median (IQR)^*b*^	62 (31–75)	76 (63–78)	0.046
Male sex, *n (%)*	24 (52)	11 (65)	0.374
Ward			
Internal Medicine, *n (%)*	21 (46)	4 (24)	0.033
Surgery, *n (%)*	14 (30)	3 (18)	
Intensive Care Unit, *n (%)*	11 (24)	10 (59)	
Comorbidities, *n (%)*	40(87)	17(100)	0.117
Chronic pulmonary diseases, *n (%)* ^*c*^	5(11)	4 (24)	0.235
Haematological malignancy, *n (%)*	1 (2)	0 (0)	0.540
Cardiovascular diseases, *n (%)* ^*d*^	15 (33)	12(71)	0.006
Neurological diseases, *n (%)* ^*e*^	9 (20)	1(6)	0.263
Gastrointestinal diseases, *n (%)* ^*f*^	16 (35)	5 (29)	0.688
Diabetes mellitus, *n (%)*	4 (9)	4 (24)	0.195
Solid tumors, *n (%)*	9 (20)	6 (35)	0.193
Chronic renal failure, *n (%)*	5 (11)	2 (12)	1.000
Previous surgery (<30 days)*, n (%)*	23(50)	9(53)	0.835
Gastrointestinal surgery, *n (%)*	5(11)	3(18)	0.671
Cardiovascular surgery, *n (%)*	7(15)	6(35)	0.080
Other surgery, *n (%)* ^*g*^	11(24)	1 (6)	0.154
Central venous catheter, *n (%)*	41(89)	15 (88)	0.920
BSI CVC-related, *n (%)*	25 (61)	11 (73)	0.460
Early central venous catheter removal, *n (%)* ^*h*^	41 (100)	11 (73)	<0.0001
Other devices, *n (%)* ^*i*^	40 (87)	17(100)	0.178
Previous invasive procedures (<72 h), *n (%)* ^*j*^	9 (20)	7 (41)	0.080
Parenteral nutrition, *n (%)*	29 (63)	14 (82)	0.143
Immunosuppressive therapy, *n (%)* ^*k*^	13(28)	5(29)	0.920
Neutropenia, *n (%)*	3 (7)	1 (6)	1.000
Septic shock, *n (%)*	1(2)	1(6)	0.470
Prior antibiotic therapy, *n (%)*	42 (91)	16 (94)	0.713
Previous antifungal therapy, *n (%)*	4 (9)	1 (6)	0.713
Concomitant bacteriemia, *n (%)*	12(26)	3(18)	0.487
Other coinfections, *n (%)* ^*l*^	28 (61)	11(65)	0.780
Appropriate antifungal therapy^*m*^	30 (65)	8 (47)	0.190
Primary azole therapy	32 (70)	9 (53)	0.219
Primary echinocandin therapy	5 (11)	2 (12)	1.000
Primary polyene therapy	1 (2)	0 (0)	1.000
None	8 (17)	6 (35)	0.129

^a^Comparisons between groups were performed using Wilcoxon rank sum test for quantitative variables and Chi-Square test (or Fisher Exact Test when expected frequencies were less than five) for qualitative variables

^b^ IQR, Interquartile range

^c^Chronic pulmonary diseases include asthma, chronic bronchitis, emphysema and lung fibrosis

^d^Cardiovascular diseases include heart failure, ischemic heart disease, endocarditis and arrhythmia

^e^Neurological diseases include Parkinson’s disease, Alzheimer’s disease and paralysis

^f^Gastrointestinal diseases include Crohn’s disease, ulcerative colitis, chronic pancreatitis and gallbladder stones

^g^Other surgery includes plastic surgery, thoracic surgery, orthopaedic surgery, urological surgery and neurosurgery

^h^ Early CVC removal was considered occurring within 48 h from blood cultures drawing

^i^ Other devices include urinary catheter, surgical drainage, cutaneous gastrostomy and tracheostomy tube

^j^ Previous invasive procedures include endoscopy and positioning of any device

^k^Immunosuppressive therapy include corticosteroids, calcineurin inhibitors and monoclonal antibodies

^l^ Other coinfections include bacterial and/or fungal infections in sites other than blood

^m^Appropriate antifungal therapy was considered when the appropriate drug with adequate dosage was started within 72 h the first blood culture performed

Table 3 shows the results of antifungal susceptibility testing to nine antifungal agents as routinely performed by the SYO method which was developed to provide an easy alternative to the CLSI procedure. With the exception of one isolate that showed to be susceptible dose dependent to fluconazole (MIC 4 μg/ml) and two isolates which were found to have non-wild type phenotype for flucytosine (MIC 1 μg/ml), all isolates showed to be fully susceptible to all antifungals according to the CLSI interpretation [15].

**Table 3:** In vitro susceptibilities of *Candida parapsilosis* complex isolates considered in this study

Antifungal agents	MIC range (μg/ml)	MIC_50_ (μg/ml)	MIC_90_ (μg/ml)	% of isolates in the indicated category according to CLSI^*a*^					
				S	SDD	I	R	WT	Non-WT
Amphotericin B	≤0.12–1	0.5	0.5	-	-	-	-	100	0
Flucytosine	≤0.06–1	≤0.06	0.25	-	-	-	-	97	3
Fluconazole	≤0.12–4	0.25	1	98.4	1.6	-	0	-	-
Itraconazole	≤0.015–0.25	0.06	0.12	-	-	-	-	100	0
Voriconazole	≤0.008–0.06	≤0.008	0.015	100	0	-	0	-	-
Posaconazole	≤0.008–0.12	0.03	0.06	-	-	-	-	100	-
Caspofungin	0.06–1	0.25	0.5	100	-	0	0	-	-
Anidulafungin	0.12–2	0.5	2	100	-	0	0	-	-
Micafungin	0.12–2	1	2	100	-	0	0	-	-

^*a*^Category was interpreted according to CLSI breakpoints as reported in ref. 15. S, susceptible; *SDD* susceptible dose dependent, *I* intermediate, *R* resistant, *WT* wild type

## Discussion and conclusions

Although *C. albicans* remains the most common fungal isolate from blood, longitudinal studies showed a trend toward an increased prevalence of other *Candida* spp. with a larger proportion of *C. glabrata* in the United States and *C. parapsilosis* in some European (i.e.: Italy or Spain) and Latin American countries [3–9]. As far, few studies focused on the specific predictors influencing the outcome of BSIs due to *C. parapsilosis* [16–18].

First, we confirmed that the mortality due to this species is somewhat lower than that reported for *C. albicans*, having found a rate of 27 % at 30 days. This figure is consistent with literature data in which the 30-day mortality due to this species ranges from 23 % to 30 % [14, 19–21].

Second, hospitalization in ICU showed to be independently associated with higher risk of mortality. Our data are in agreement with a large study showing a mortality rate increasing from 29 % to 47 % in patients with BSIs due to *Candida* spp. hospitalized, respectively, in non-ICU and ICU wards [22]. ICU stay would represent a surrogate marker of illness severity thereby facilitating the poor outcome of this population group.

Third, we showed that early CVC removal is protective in BSIs due to *C. parapsilosis.* Although the impact of CVC management has been extensively investigated on the outcome of patients with candidemia, few studies have examined this issue across individual *Candida* species. Two recent studies showed that CVC removal exerted a protective effect on the outcome of candidemia due to *C. parapsilosis* [14, 20]. Since this species is characterized by a high propensity to develop biofilms on intravascular devices, their early removal play a fundamental role in determining the outcome of infection.

Fourth, we found that the outcome was not influenced by an appropriate antifungal therapy. Despite this finding is somewhat divergent from that reported by the current literature [23–25], there is a paucity of data considering the infection outcome of individual *Candida* species. In this regard, there are two important factors to consider. First, being *C. parapsilosis* less pathogenic than other *Candida* species (i.e.: *C. albicans* and *C. tropicalis*) [17], BSIs due to this entity could be more deeply affected by a correct general management (i.e.: prompt removal of any central line) rather than an early therapeutic intervention. Second, we defined appropriate antifungal therapy as the appropriate drug with adequate dosage started within 72 h from the first blood culture performed. Since *C. parapsilosis* has a time to positivity of blood cultures longer than those reported for *C. albicans* and *C. tropicalis* [26], preliminary blood culture showing the growth of yeast-like fungal pathogen could be delayed thereby determining an initial, although adequate, antifungal treatment after this time interval.

Interestingly, we found that primary antifungal treatment (i.e.: triazoles or echinocandins) did not influence the outcome of *C. parapsilosis* BSIs. Although, this *Candida* species possesses a natural low susceptibility profile to echinocandins [12] and there is still a debate on the use of these molecules in infections caused by this species, either randomized or not-randomized clinical trials have shown not significant differences in the success rates between arms [27–30]. Our data, although with a limited number of patients, corroborated these findings.

The present study have some limitations. First, being a single-center study, the number of patients considered is low. This feature has certainly weakened the statistical power of the study. Nevertheless, we have made all attempts to collect and analyze as many as clinical data as possible to reveal useful information for the management of patients infected with this *Candida* species. Second, being a retrospective study encompassing several departments and medical disciplines over a five years period, there was not a univocal management of each individual case. In this respect, serial follow-up blood cultures were not systematically performed and we were unable to include important parameters, other then 30-day mortality, such as the persistence of positive blood cultures after the initiation of antifungal therapy.

In conclusion, our study shows that candidemia due to *C. parapsilosis* complex, the second most commonly causative agent of yeast BSIs in our center, is characterized by a non-negligible mortality at 30 days. While an early CVC removal is associated with significant reduced mortality, the type of primary antifungal therapy does not influence the outcome. Further prospective studies including higher number of patients are needed to corroborate these findings.

## Abbreviations

BSIs, bloodstream infections; CBP, clinical breakpoints; CLSI, Clinical and Laboratory Standards Institute; CVC, central venous catheter; HR, hazard ratio; ICUs, Intensive Care Units; IDSA, Infectious Disease Society of America; MALDI-TOF, Matrix Assisted Laser Desorption/Ionization Time of Flight Mass Spectrometry; MICs, minimum inhibitory concentrations; Q1-Q3, interquartile range; spp, species; h, hours.; SPSS, Statistical Package for the Social Sciences; SYO, SensitreYeastOne colorimetric plate
